# Anti-predation behavior resolves sexual conflict over mating rate and duration in a protandric-simultaneous hermaphrodite

**DOI:** 10.7717/peerj.21804

**Published:** 2026-10-08

**Authors:** J. Antonio Baeza

**Affiliations:** 1 Department of Biological Sciences, Clemson University, Clemson, SC, United States of America; 2 Smithsonian Marine Station at Fort Pierce, Smithsonian Institution, Fort Pierce, FL, United States of America; 3 Departamento de Biologìa Marina, Universidad Catolica del Norte, Coquimbo, Chile

**Keywords:** Sexual conflict, Mixed sexual system, Hermaphrodite, Sex allocation, Mating costs

## Abstract

The reproductive interests of males and females rarely coincide, and thus, sexual conflict should be widespread in sexual species and favor the evolution of traits that bias the outcome of mating interactions towards the participant’s own interests. Sexual conflict theory has achieved considerable sophistication since its formal proposal. Yet, the evolutionary implications of this process in species other than those with separate sexes remain ill-explored. I examined sexual conflict in *Lysmata wurdemanni*, a shrimp that exhibits protandric simultaneous hermaphroditism, a peculiar sexual system where individuals invariably mature and reproduce as male first before becoming simultaneous hermaphrodites. In *Lysmata wurdemanni*, exacerbated mating costs for female-role individuals included death by too-eager-to-mate male-role individuals and resulted in the unprecedented employment by female-role hermaphrodites of anti-predatory tactics (escape response consisting of a backward tail flipping that rapidly propel shrimp away from danger) to actively resist harassment and to exert control over copula termination. Sexual conflict does occur in species featuring unconventional sexual systems and likely has implications for sex allocation patterns.

## Introduction

The reproductive interests of males and females rarely coincide, and thus, sexual conflict should be widespread in sexual species (Parker, 1979; Arnqvist & Rowe, 2005). Sexual conflict can be resolved *via* persuasion, coercion and/or force, with unbalances in power, information, and payoffs of alternative strategies as key drivers of the outcome (Arnqvist & Rowe, 2002; Arnqvist & Rowe, 2005). Sexual conflict is expected to favor the evolution of traits that bias the outcome of sexual interactions towards the participant’s own interests (Rowe & Day, 2006; Crespi, 2007). For instance, in gonochoric species (with separate sexes), sexual conflict over mate-guarding duration, copula duration, and/ or mating rates, among others, might result in antagonistic sexual selection and escalate into runaway evolutionary arm races between the sexes that ultimately determines the exaggeration of external structures which confer a reproductive advantage to one sex over the other (Arnqvist & Rowe, 2002; Arnqvist & Rowe, 2005). A now classic example of an arm race between males and females fueled by sexual conflict occurs in *Gerris incognitus* where males have evolved traits to forcefully grasp and mate with females, while females have evolved counter-adaptations (*i.e.,* abdominal spines and shields) to fend off unwanted mating (Perry & Rowe, 2012). Arm races fueled by sexual conflict over the mating sexual role also occur in simultaneous hermaphrodites with reciprocal insemination (mating individuals act simultaneously as sperm recipients and donors) and where cryptic female choice is relevant (Arnqvist & Rowe, 2005; Koene & Schulenburg, 2005; Chase & Blanchard, 2006; Anthes, Schulenburg & Michiels, 2008). In this case, the evolutionary by-product is the exaggeration of manipulative male organs and female organs involved in sperm selection (Koene & Schulenburg, 2005; Chase & Blanchard, 2006; Anthes, Schulenburg & Michiels, 2008).

Sexual conflict is a thriving research field, and this theory has achieved considerable sophistication since its formal proposal (Arnqvist & Rowe, 1997; Arnqvist & Rowe, 2002; Arnqvist & Rowe, 2005; Parker, 1970; Parker, 1979; Rice, 1996; Trivers, 1972, also see Arnqvist & Rowe, 2005 and references therein). The nature, details and evolutionary implications of sexual conflict are increasingly understood in gonochoric and simultaneously hermaphroditic species but remain ill-explored in species featuring other less common sexual systems (Arnqvist & Rowe, 1997; Arnqvist & Rowe, 2002; Arnqvist & Rowe, 2005; Cutter, Morran & Phillips, 2019; Parker, 1970; Parker, 1979; Rice, 1996; Schmitt, Anthes & Michiels, 2007; Schärer & Janicke, 2009; Ting et al., 2014; Trivers, 1972). Nonetheless, sexual conflict might have most relevant consequences at the individual, population and species level in organisms with rare gender expression patterns if *e.g.*, mating costs are unbalanced or other reproductive interests differ for individuals playing the different sex roles (*e.g.*, Chasnov, 2010; Benvenuto & Weeks, 2012).

Recently, studies reporting species with unusual sexual systems have increased substantially (*e.g.*, bi-directional sex change in goby fishes—St. Mary, 1997; Munday, Caley & Jones, 1998: males coexisting with simultaneous hermaphrodites in androdioecious clam shrimps—Zucker, Cunningham & Adams, 1997). One of the most enigmatic mixed sex allocation patterns is protandric simultaneous hermaphroditism (*sensu* Bauer & Holt, 1998) in which individuals consistently mature and reproduce initially as males, and later in life, become functional simultaneous hermaphrodites. This sexual system is also called ‘adolescent protandry’ (*sensu* Ghiselin, 1974). Protandric simultaneous hermaphroditism has been confirmed in a polychaete worm (*Ophryotrocha diadema*—Premoli & Sella, 1995), in a land snail (*Achatina fulica*—Tomiyama, 1996), and in marine shrimp species from the genus *Lysmata* (Bauer, 2000; Baeza, 2009), *Exhippolysmata* (Braga et al., 2009; Baeza et al., 2010), *Parhippolyte* (Onaga, Fiedler & Baeza, 2012), and *Salmoneus carvachoi* (Oliveira et al., 2015; Oliveira et al., 2024). Research in these protandric simultaneous hermaphrodites is warranted because it provides opportunities to test predictions fundamental to sexual conflict theory and insights on the universality of this process in shaping mating interactions.

In the protandric simultaneous hermaphroditic shrimp *L. wurdemanni* mating costs are unbalanced for individuals playing the different sex roles. Reproduction of hermaphrodites as females occurs during or seconds after moulting and involves insemination by male-role shrimp and subsequent spawning and brooding of fertilized eggs (Bauer & Holt, 1998). Males and hermaphrodites can mate as males frequently, but mating is not reciprocal: *i.e.,* a newly molted and soft-body pre-spawning hermaphrodite copulates as a female, with a male or another hermaphrodite that acts only as male (donate sperm) at that time (Bauer & Holt, 1998). Mating comprises interception of female-role shrimp, forceful retention and external sperm transfer to female-role by male-role shrimp (Baeza, 2007a). That female but not male-role shrimp mate with a soft-body condition and the scrambles among male-role shrimps for access to females (Baeza, 2007a) set the stage for sexual conflict: mating costs *e.g.*, body injuries and/or death are potentially severe for female-role but negligible for male-role shrimps. Supporting the notion of unbalanced mating costs, detailed observations of mating interactions have revealed that female-role shrimp intensively use an anti-predatory ‘escape response’ (*i.e.,* a backward ‘tail’ [= abdominal] flipping that rapidly propel shrimp away from danger) (Baeza, 2007a; Baeza, 2007b; Baeza, 2007c; Baeza, 2009). However, it is not known if this escape response is routinely used by female-role shrimps to resist harassment by male-role shrimps before molting, to exert control over copula termination, and to avoid further male advances after insemination.

In this study, I examined the nature of conflict and its resolution in the protandric simultaneous hermaphrodite *L. wurdemanni*. In gonochoric species, males are known to harass females to a greater extent when faced with extra competition (Le Galliard et al., 2005). Similarly, in *L. wurdemanni*, mating costs are expected to increase with mating group size, due to augmented male-male competition (Baeza, 2007a; see also Le Galliard et al., 2005). Thus, mating group size was experimentally manipulated in the laboratory to increase the extent of conflict during copula and to reveal costs that female-role shrimp might suffer during mating. The same experimental manipulation permitted testing whether shrimps used stereotyped behaviors (*e.g.*, the escape response) to routinely resist harassment before molting, to exert control over copula termination, and to avoid male advances after insemination. Specifically, I examined the null hypothesis of no effect of mating group size in the “escape behavior” and copula duration of female-role hermaphrodites when maintaining large or small shrimp aggregations in the laboratory and recording the mating behavior of pre-spawning hermaphrodites close to molting and reproducing in the female role. Given the potentially exceptional mating costs, especially at high densities, female-role shrimp were predicted to intensify avoidance behavior (*i.e.,* tail flips) and reduce copula time to ameliorate these costs at high compared to low rearing densities.

## Material and Methods

### The model system

Shrimps from the genus *Lysmata* are rare among crustaceans because of their enigmatic sexual system. All species so far studied are protandric simultaneous hermaphrodites, in which individuals consistently mature and reproduce initially as males, and later in life, after a single transitional instar (*i.e.,* hereafter transitionals), become functional simultaneous hermaphrodites (*i.e.,* hereafter hermaphrodites) (Bauer & Holt, 1998; Fiedler, 1998; Baeza, 2009). Among them, *Lysmata wurdemanni* (Caridea: Hippolytidae) was used as model for the study of sexual conflict because of the number of studies accumulated in this shrimp (Bauer & Holt, 1998; Bauer, 2000; Bauer, 2002a; Bauer, 2002b; Bauer, 2006; Baeza & Bauer, 2004; Baeza, 2006; Baeza, 2007a; Baeza, 2007b; Baeza, 2007c; Baeza, 2009; Baeza et al., 2009; Rhyne & Lin, 2006).

In *L. wurdemanni*, males bear typical caridean male secondary characters and are only capable of reproducing as males, though their gonads are ovotestes (Bauer & Holt, 1998). Males later attain the female sex function developing into functional simultaneous hermaphrodites. Males transition to simultaneous hermaphrodites at a range of body sizes and environmental, including social, cues drive the timing of sex-phase change (Baeza, 2007b; Baeza & Bauer, 2004). As described in Baeza (2018), externally, hermaphrodites resemble females of caridean gonochoric species, brooding embryos in an abdominal chamber (Bauer & Holt, 1998; Baeza, 2007a). However, hermaphrodites retain testicular tissue, male ducts and gonopores, and thus can and do reproduce as both male and female (Bauer & Holt, 1998). Hermaphrodites can mate as males frequently, but copulation with another hermaphrodite is not reciprocal: *i.e.,* a newly molted pre-spawning hermaphrodite copulates as a female, with a male or another hermaphrodite that acts only as male at that time (Bauer & Holt, 1998). A sperm mass is attached to the underside of the pre-spawning hermaphrodite by a mating partner during copulations that last a few seconds (Bauer & Holt, 1998). Sperm from a mating is used to fertilize only eggs released during the spawning event that occurs 2–3 h later. There is no long-term sperm storage in this shrimp. After becoming hermaphrodites, individuals do not revert to their initial sexual condition, and no self-fertilization has been demonstrated (Bauer, 2002a). In previous studies, the adaptive value of having an early protandric phase (Baeza, 2006), the conditions determining the timing at which males mature as hermaphrodites (Baeza & Bauer, 2004; Baeza, 2007a; Baeza, 2007b) and the factors affecting the sex allocation pattern of the simultaneously hermaphroditic phase of this shrimp have been examined (Baeza, 2007c).

### Collection and maintenance of shrimp

Collection and maintenance of shrimp occurred as previously described in Baeza (2018). Specifically, for laboratory experiments, individuals of *L*. *wurdemanni* were collected from the rock jetty at Mustang Island, Port Aransas, Texas (27°50′N, 97°03′W) during Spring and Summer months (May to August) and transported in plastic bags filled with bubbling water to the wet laboratory. In the laboratory, individuals were maintained prior to the experiments in 80-L or 114-L recirculating aquaria at a water temperature of 21–25 °C, 34–35 ppt salinity, and a light:dark cycle of 14:10 h. Shrimp were fed daily (*ad libitum* Wardley Shrimp Pellets, Hartz Mountain Corp., Secaucus, NJ).

### Sexual conflict: detecting costs and female behavioral response

I examined the null hypothesis of no effect of mating group size in the “escape behavior” and copula duration of female-role hermaphrodites when maintaining large or small shrimp aggregations in the laboratory and recording the mating behavior of ‘parturial’ hermaphrodites. ‘Parturial’ hermaphrodites are pre-spawning hermaphrodites close to molting and reproducing in the female role (Bauer, 2002a). Small mating groups consisted of two shrimps, one large hermaphrodite and one small male. Large mating groups comprised shrimps with a size and sex-morph proportion similar to that found during main reproductive season in the field; the relative proportion of small males (shrimps < 6.0 mm carapace length, CL) to large males (shrimps > 6.0 mm CL) to small hermaphrodites (shrimps < 9.0 mm CL) to large hermaphrodites (shrimps > 9.0 mm CL) in the field is 4:3:2:1, respectively (calculated from data in Bauer, 2002b). This relative proportion was emulated in the laboratory by placing combinations of 10 shrimps of different sizes and sexual phases (4:3:2:1) into 20 L aquaria (40  ×  20  × 25 cm).

Data were collected as previously described in Baeza (2018). Specifically, in each replicate, after an initial acclimatization period of 2 h, one ‘parturial’ hermaphrodite was introduced into an aquarium already containing small or large mating groups. Molting and the subsequent number of inseminations experienced by this ‘parturial’ hermaphrodite were counted in each of 28 and 31 replicates from the two treatments, respectively. Recording of parturial shrimp behavior was conducted with time-lapse video (GYRR model TLC1400) at a speed of 5 frames per second (24–h recording mode). A Cohu CCD surveillance video camera equipped with a 6–mm lens sensitive to both low–intensity white and infrared light was used. Fluorescent and infrared (880 nm) lamps provided day and night illumination, respectively. Videotapes were analyzed counting the number of escape responses (tail flips) performed by the female-role shrimp every time period of 5 s during the first 20 s before molting, and the first 20 s after mating. The number of escape responses between molting and mating were also counted for those replicates in which insemination occurred after the parturial shrimp molted.

The rationale of the experiment above was maximizing costs during copula for female-role shrimp. Increases in mating group size translates into greater chances for female-role hermaphrodites that have just molted of suffering physical injuries during mating if being simultaneously detected and intercepted by a larger number of male-role shrimps (Le Galliard et al., 2005; Baeza, 2007a; Baeza, 2007b).

During the experiment, copula was observed while female-role shrimps were molting or seconds after molting, when the female-role hermaphrodite liberated itself from the molted carcass and began swimming. Thus, I tested for the effect of mating group size (small *versus* large) and moment of insemination (when the parturial shrimp was molting *versus* swimming seconds after molting) on the number of backward escape responses (n tail flips sec^−1^) using a two-way repeated measurements ANOVA. The circularity assumption of this test was violated (Mauchly’s criterion for sphericity; *P* < 0.001). Therefore, I used the adjusted F statistics instead of the inflated univariate F statistics in order to decrease the probability of a type I error for the within-subject factor (*i.e.,* time periods; Tabachnick & Fidell, 2007). Lastly, an *a posteriori* contrasts Profile analysis was conducted to compare the intensity of tail flips among adjacent time periods (5 s).

In the experiment above, copula duration (between the parturial hermaphrodite and the second shrimp serving as a male) was recorded for each replicate. I tested the hypothesis of no effect of group size and moment of insemination with a two-way analysis of variance (ANOVA) in SPSS 17.0. Assumptions of normality and homocedasticity of the data set were examined and found to be satisfactory.

## Results

### Sexual conflict: cost and female behavioral response

By experimentally manipulating mating group size, I increased the degree of conflict during copulation and identified potential costs incurred by female-role shrimp during mating. Notwithstanding mating group size, female-role shrimp invariably mated once with a single male-role shrimp during the experiment (100%, *N* = 59 replicates). Too-eager-to-mate male-role shrimp killed female-role hermaphrodites in 6.9% (2 out of 31 replicates) of the sexual interactions at high densities but none at low densities (0 out of 28 replicates). The small number of replicates in which female-role shrimps were killed precluded statistical comparison between treatments. Death of female-role shrimp occurred when grasped, simultaneously, by various male-role shrimp and during overly long chasings in which insemination was repeatedly avoided with intense use of tail flipping by parturial hermaphrodites. Potentially harming interactions were seen during many mating interactions, with male-role shrimps pooling pereopods (walking legs) or grasping female-role shrimps while attempting inseminations.

Experimental manipulation of mating group size additionally permitted to confirm that female-role shrimps routinely use an anti-predatory “escape response” (backward tail flipping that rapidly propel shrimp away from danger) to resist harassment before moulting, to exert control over copula termination, and to avoid male advances after insemination. In *L. wurdemanni*, anti-predator behaviors (escape response) were more frequently employed at high than at low mating group sizes and immediately before molting and after copula. Time affected the intensity of the backward escape response during mating (Wilks’ Lambda: *F* = 22.65, *df* = 8,46, *P* < 0.001) and a profile analysis indicated that the number of tail flips increased seconds before molting but particularly seconds after mating (Fig. 1). As expected, the escape response was also more frequently employed at large than at small mating group sizes (*F* = 23.65, *df* = 1,53, *P* < 0.001; Fig. 1A) but was not affected by the timing of insemination (*F* = 0.156, *df* = 1,53, *P* = 0.694). No interaction between group size and moment of molting was recorded (*F* = 0.027, *df* = 1,53, *P* = 0.871).

**Figure 1:**
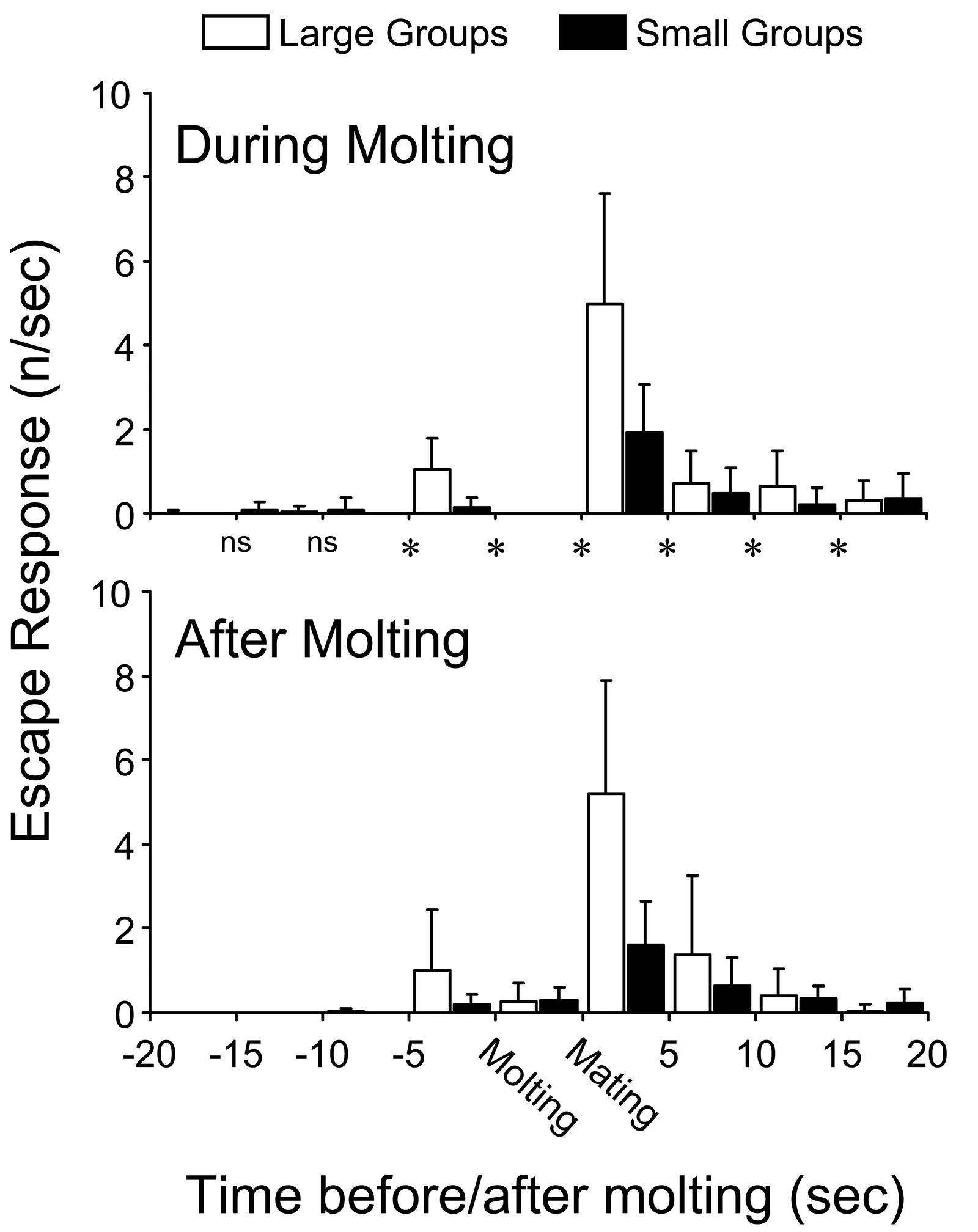
The number of escape responses per focal female-role hermaphrodite exposed to low and high density treatments before and after molting and mating. Asterisks (*) indicate significant differences among adjacent time periods as indicated by a profile analysis.

Copula, that was usually terminated with an escape response by the parturial hermaphrodite, lasted shorter at large than at small group sizes (two-way ANOVA: *F* = 6.28, *df* = 1, 53, *P* = 0.0153) but was not affected by the timing of insemination (*F* = 2.04, *df* = 1, 53, *P* = 0.1588). The interaction term was not significant (*F* = 0.14, *df* = 1,53, *P* = 0.7051; Fig. 2).

**Figure 2:**
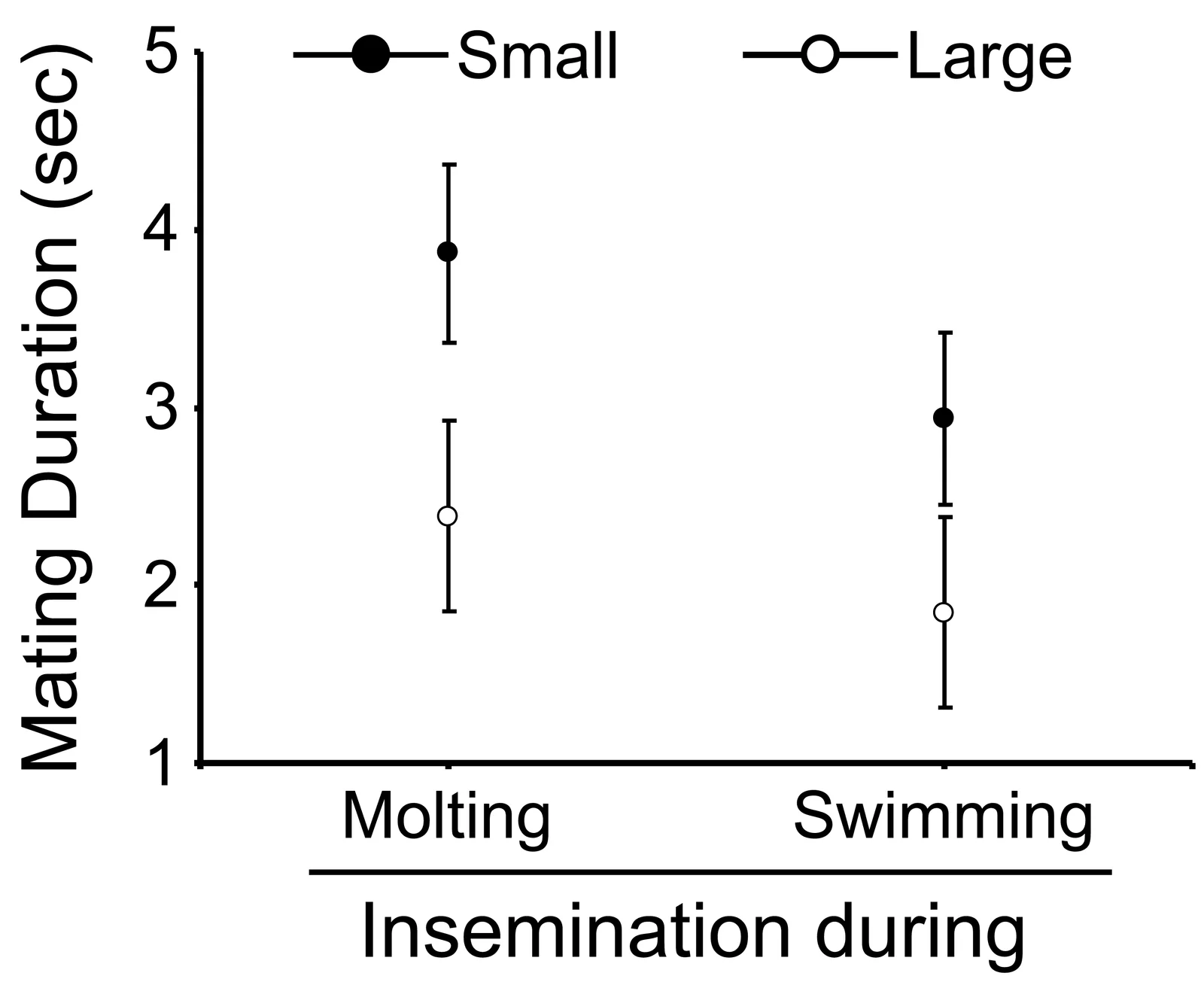
Copula duration (sec) female-role hermaphrodite exposed to low and high density treatments before and after molting and mating.

## Discussion

In *L. wurdemanni*, female-role shrimp do suffer costs during mating interactions; *i.e.,* too-eager male-role shrimp killed female-role hermaphrodites during sexual interactions in large mating groups. Although logistic constraints did not permit to measure physical injury, it is likely that the latter might be an additional cost experienced by parturial hermaphrodites during mating interactions. It could be argued that food constraints might have triggered cannibalistic behaviors that superseded the mating drive in male-role shrimps during the experiment. Nonetheless, food was provided *ad libitum* to shrimps for a long period before and during the experiment. At present, the information suggests that killing of female-role hermaphrodites by male-role shrimps is not an experimental artifact, and thus, mating costs are exceptional in *L. wurdemanni*. Female killing during sexual encounters is most rare but has been increasingly reported during the last decades (Parker, 1979; Izzo et al., 2012; Smit, 2025). Also, female harming during mating and diminished survival after forcible insemination attempts are increasingly reported in vertebrate and invertebrate taxa with gonochoric sexual systems (Reale, Bousses & Chapuis, 1996; Crudgington & Siva-Jothy, 2000; Stutt & Siva-Jothy, 2001; Arnqvist & Rowe, 2002; Le Galliard et al., 2005; Hotzy & Arnqvist, 2009; Schärer & Janicke, 2009). I argue that future studies need to address additional costs of mating for female-role shrimp in *L. wurdemanni*. Costs might include damage or loss of appendages, like pereiopods and antenna, which are vital for feeding, swimming, crawling, and sensing the environment in shrimp (Bauer, 2023). Laboratory experiments coupled or not with population dynamics studies can be designed to determine how often damage to appendages happens during copula during the mating season. In other species in which sexual conflict has been reported, costs other than mortality include direct physical harm (*i.e.,* injuries) and indirect detrimental effects (Smit, 2025). Indirect costs include, among others, decreased female longevity as well as diminishing reproductive pace, fecundity, and offspring ‘genetic quality’ (Smit, 2025 and references therein).

Given the exceptional mating costs, female-role shrimps were predicted to routinely used stereotypes behaviors to ameliorate these costs. Supporting the notions above, recording of shrimp behavior confirmed that female-role individuals do routinely use an anti-predatory “escape response [tail flip]” to resist harassment before molting, to exert control over copula termination, and to avoid male advances after insemination. Although, female physical resistance before mating occurs in several gonochoric (separate sexes) species (Arnqvist & Rowe, 2002 and references therein), to the best of my knowledge, this is the first species reported to use, intensively, anti-predatory tactics during sexual encounters. Furthermore, female-role shrimps were predicted to intensify predator avoidance behaviors and reduce copula time to ameliorate exacerbated mating costs in large mating groups. As expected, anti-predator behaviors were more frequently employed when in large compared to small mating group sizes. Similarly, copula, that was usually terminated with an escape response, was shorter at large than at small mating group sizes. These results suggest that female-role shrimps do employ stereotyped anti-predatory tactics to resolve conflict over mating rate and duration. The highly unbalanced mating costs in *L*. *wurdemanni* have favored the recruitment by female-role shrimps of a most conspicuous anti-predatory tactic to serve as a counter-adaptation to forcible male insemination. These results further imply that sexual conflict is pervasive, occurring in species with unconventional sexual systems (*e.g.*, protandric simultaneous hermaphroditism) and suggests that mating rate is a sexually antagonistic shared trait and the escape response represents a most conspicuous sexually antagonistic female trait in *L. wurdemanni* (see Rowe & Day, 2006).

In the following I discuss the possible consequences of intensive sexual conflict in the studied species.

### Sexual conflict consequences as it relates to sex allocation in *Lysmata wurdemanni*

Sexual conflict shapes mating interactions in *L. wurdemanni*. Sexual conflict might also indirectly affect other phenotypic traits in this shrimp. Specifically, sexual conflict, through its effect on female behavior, might affect the optimal amount of resources that hermaphroditic individuals invest to the sex functions (*i.e.,* sex allocation). Notwithstanding density, female-role shrimp invariably mated once with a single male-role shrimp during our experiment, indicating, on the one hand, that heavy mating costs favours monandry and probably preclude females from gaining access to indirect benefits from males (Cameron, Day & Rowe, 2003). On the other hand, this monoandrous behavior is expected to bias sex allocation of hermaphrodites towards the female function (ovary mass), given the absence of sperm competition (Charnov, 1982; Leonard, 2006; Baeza, 2007c). In agreement with this prediction, sex allocation is highly female biased in hermaphrodites (*i.e.,* the average ratio [± sd] of female to male dry reproductive mass is 117.9 (± 38.9) in *L. wurdemanni*—Baeza, 2007c). Elevated mating costs might also explain preference for small sexual partners previously demonstrated for female-role shrimps (Baeza, 2007c), a choice that contrasts with the rather standard preference for large males widely reported for females of gonochoric species (Darwin, 1871; Shuster & Wade, 2003). Large mating partners might increase the risk of suffering injuries (or even death) in *L. wurdemanni*, as reported before for other gonochoric arthropods (Pitnick & García-González, 2002). Thus, female-role hermaphrodites might prefer to mate with small rather than large mating partners so to avoid the potentially high costs of mating. Also, hermaphrodites invest energy to the male function, measured as ovotestes mass, more than proportionally when small than large to profit from male mating opportunities that are greatest at small body sizes (Baeza, 2007c). Thus, size-dependent sex allocation might be considered another indirect outcome of sexual conflict through its effect on female mating preferences (see Baeza, 2007c; Baeza, 2010). Additional studies are needed to understand the effect of sexual conflict on the optimal sex allocation of protandric simultaneous hermaphrodites.

### Sexual conflict consequences as it relates to sex ratio and population dynamics in *Lysmata wurdemanni*

Sexual conflict shapes mating interactions and might drive optimal sex allocation in protandric simultaneous hermaphrodites as discussed above. It could be argued, as well, that sexual can affect sex ratio and population dynamics in *L. wurdemanni*. That female-role shrimp died in large but not in small mating groups and the more intense use of escape responses by female-role shrimps in large compared to small mating group sizes suggests that mating costs experienced by female-role hermaphrodites increase at high densities. Considering the above, it could be expected a decline in the relative abundance of hermaphrodites throughout the breeding season of the studied species concomitantly with increasing shrimp density due to male recruitment to the population. Supporting this prediction, sex ratio has been shown to increase sharply and remained greatly skewed toward males while body size of hermaphrodites consistently plummeted throughout the breeding season in *L. wurdemanni* (Baldwin & Bauer, 2003). Although, the proportion of males changing to hermaphrodites remained somewhat constant throughout the reproductive period, the population dynamics of *L*. *wurdemanni* indicated that at the peak of and late during the breeding season, shrimps mature as hermaphrodites early in life and die soon after (Baldwin & Bauer, 2003). This sharp decline in hermaphrodite abundance during the reproductive season might alternatively be caused by a higher mortality among hermaphrodites than males caused by reproductive exhaustion (Berglund & Rosenqvist, 1986; Lindstrom, 2001), higher susceptibility to parasites (Calado, Bartilotti & Narciso, 2005), or increased predation risk (Berglund & Rosenqvist, 1986). However, small groups of hermaphrodites maintained in the laboratory with conditions emulating those in the field during the breeding season live for months reproducing continuously (Baeza, 2007b), no presence of parasites that might have impacted survivorship of hermaphrodites were observed during dissections, and experiments have demonstrated that risk of mortality due to the main natural fish predator of this shrimp (*i.e.,* the fish *Scartella cristata*) is not sex-dependant and decreases with increasing body size of hermaphrodites, and not the other way around (Baeza, 2007c). Although, an effect of reproductive exhaustion, parasites, or increased predation risk on the population dynamics of *L. wurdemanni* is certainly plausible (and most likely present in the field), experimental results suggests and additional explanation for field observations; increased mortality experienced by female-role shrimp plays a role in explaining the decline in hermaphrodite numbers through the breeding season. One implication from this result is that sex ratios in natural populations might not be an optimal trait (see Charnov, 1982) but instead the immediate outcome of sexual conflict in *Lysmata wurdemanni*. Studies on sex ratio within this genus of protandric simultaneous hermaphrodites is warranted as it will permit testing predictions central to sex allocation theory and explore the interaction between sexual conflict and optimal resource allocation in explaining population-level sex allocation patterns in species with mixed sexual patterns (see below).

This study suggests that highly unbalanced mating costs in a protandric simultaneously hermaphroditic shrimp has favored the recruitment and use of an anti-predatory “escape response” in female-role individuals, a most conspicuous counter-adaptation to forcible male insemination. Extreme mating costs for females have resulted in exacerbated and unprecedented conflict resolution and important indirect individual-level effects. These results fit well with a highly asymmetric inter-sexual arms race scenario (Dawkins & Krebs, 1979) resulting in the over-exaggeration of female behavioral resistance driven by exacerbated mating costs. Importantly, sexual conflict might impact, directly and indirectly, relevant individual traits (mating behavior and sex allocation) in *L. wurdemanni*.

Whether the consequences of antagonistic sexual selection are evident at the interspecific level in shrimps from the genus *Lysmata* remain to be addressed. In gonochorists, sexual conflict might escalate into runaway evolutionary arms races between the sexes with the exaggeration of arms in one sex (*i.e.,* grasping structures in males) and counter-adaptations in the other sex (Dawkins & Krebs, 1979; Arnqvist & Rowe, 2002; Arnqvist & Rowe, 2005). In contrast to that predicted for gonochoric species, such an arm race with exaggeration of external morphological traits might not occur in protandric simultaneously hermaphroditic shrimps from the genus *Lysmata* because there is no opportunity for manipulation of the sexual partner given the absence of exchange of substances other than sperm during copula and the lack of ejaculate receptacles in female-role individuals (Arnqvist & Rowe, 2002; Arnqvist & Rowe, 2005). Furthermore, the hermaphroditic condition is expected to limit the development of external sex-specific traits in the absence of sperm competition and cryptic female choice (Leonard, 2006). Instead, an evolutionary arms race might occur at the behavioral level in this shrimp. Male-role shrimp are very eager to mate and copula, controlled by female-role shrimp, last shorter in high density (*L. boggessi*) than in low density species (*L. amboinensis*) (Zhang, Rhyne & Lin, 2007). Whether female-role shrimp from high density species are more reluctant to mate and employ the escape response more intensively during mating than low density species remains to be addressed. Overall, the evolutionary by-product of sexual conflict does not necessarily need to be the exaggeration of body traits (see Arnqvist & Rowe, 2005), especially in species with sexual systems that include simultaneously hermaphroditic individuals where partner manipulation is constrained.

It has been shown that female-role hermaphrodites of *Lysmata wurdemanni* mate during or within minutes after molting and the experimental data suggest that, during this period, their soft-bodied condition makes them particularly vulnerable to mating-related harm, including mortality caused by male-role shrimp. This period of vulnerability is not unique to *L. wurdemanni* but is characteristic of caridean shrimps more broadly, in which females become sexually receptive following molting (Bauer, 2023). Thus, mating costs like those documented here may occur in other caridean shrimps, irrespective of their sexual or mating systems. However, detailed descriptions of copulation and mating interactions remain scarce for caridean shrimp, and comparable studies are lacking for other species of *Lysmata* (Baeza, 2018). In *Rhynchocinetes uritai* (fam. Rhynchocinetidae), a strictly protandric hermaphrodite, limited laboratory observations indicate that some females “jump away” immediately after copulation (Bauer & Thiel, 2011), suggesting a possible post-copulatory escape response. In contrast, in the congeneric gonochoric species *R. brucei*, copulation is reportedly terminated by males (Thiel, Chak & Dumont, 2010). Further comparative studies are needed to determine the prevalence and functional significance of such escape responses and, more broadly, to assess the nature and extent of sexual conflict across caridean shrimps.

In summary, in this study, I examined the null hypothesis of no effect of mating group size in the “escape behavior” and copula duration of female-role hermaphrodites. For that purpose, large or small shrimp aggregations were experimentally established under laboratory conditions, and the mating behavior of pre-spawning hermaphrodites close to molting and reproducing in the female role were recorded. It was predicted that female-role hermaphrodites will intensify avoidance behavior (*i.e.,* tail flips) and reduce copula time especially at high compared to low rearing densities to ameliorate the potentially high mating costs compared at high when compared to low rearing densities. The experimental results support predictions and suggest that mating costs are exceptional in the studied species and include mortality due to too-eager-to-mate females. I argue in favor of additional natural history and experimental studies to continue improving our understanding of sexual conflict and mating dynamics in species with mixed as well as conventional sexual systems.

### Outlook

The present results in *L. wurdemanni* can be interpreted as supporting both the general argument of ‘conflict-of-interest’ between males and females formulated by Trivers (1972) and Parker (1979) and the more explicitly antagonistic perspective emphasized in later studies by Rice (1996), Arnqvist & Rowe (2002) and Arnqvist & Rowe (2005), among others. In the classical framework proposed by Trivers (1972) and Parker (1979), sexual conflict arises whenever the reproductive outcome that maximizes fitness differs between mating partners. In *L. wurdemanni*, the contrasting behavior of male- and female-role shrimp is consistent with sex-specific divergent reproductive interests: male-role shrimp actively pursue, grasp, and attempt to inseminate recently molted hermaphrodites, while female-role individuals actively and intensively employed a ‘escape responses’ before, during, and immediately after copulation exerting control over mating duration and termination. The observed male- and female-role sexual interactions in *L. wurdemanni* can also be interpreted as supporting the sexually antagonistic framework developed by Rice (1996), Arnqvist & Rowe (2002) and Arnqvist & Rowe (2005) in which greater emphasis is placed on traits (including behaviors) that can increase the reproductive success of one sex while imposing fitness costs on the other (plus counter-adaptations that may evolve). In the studied species, increased mating-group size intensified female-role escape behavior, copulations were generally terminated by female-role individuals, and excessive male-role persistence occasionally resulted in the death of recently molted hermaphrodites. These elements characterizing the mating system of *L. wurdemmani* resembles the ‘persistence–resistance scenario’ proposed by Rice (1996), Arnqvist & Rowe (2002) and Arnqvist & Rowe (2005). The female-role shrimp escape response observed in *L. wurdemanni* can be interpreted as a behavioral mechanism that shifts the outcome of mating interactions toward the female-role optimum and reduces the potentially severe costs imposed by to-eager-to-mate male-role shrimp. Furthermore, female-resistance behaviors (*i.e.,* escape response) can be interpreted as a form of female-role mate choice in *L. wurdemanni* (see Cordero & Eberhard, 2003; Eberhard & Cordero, 2003). This study supports the notion that sexual conflict theory is a most relevant evolutionary process beyond gonochoric species. I argue that *L. wurdemanni* can be used as a model system to continue understanding the details of the sexual dynamics, including sexual conflict, in species with mixed sexual systems.

##  Supplemental Information

10.7717/peerj.21804/supp-1 Supplemental Information 1 Raw Dataset

## Additional Information and Declarations

### Data Availability

The following information was supplied regarding data availability: The raw data are available in the Supplemental File.
